# How mixed ownership affects investment efficiency? evidence from state-owned enterprises in China

**DOI:** 10.1371/journal.pone.0306190

**Published:** 2024-06-25

**Authors:** Xiaoping Huo, Yunpei Zhao, Zhihao Dong

**Affiliations:** 1 School of Economics and Management, Guangxi Normal University, Guilin, China; 2 Business School, Guilin University of Technology, Guilin, China; 3 Education Department of Guangxi Zhuang Autonomous Region, Key Laboratory of Digital Empowerment Economic Development (Guangxi Normal University), Guilin, China; Lincoln University, NEW ZEALAND

## Abstract

The inefficiency observed in investment within state-owned enterprises presents a significant practical challenge that can affect the sustainable development of China’s economy. To address this issue, this study comprehensively explores the intricate mechanisms underlying the governance implications of mixed ownership on the investment efficiency of listed companies. Drawing on unbalanced panel data encompassing Shanghai and Shenzhen Stock Exchange A-share listed companies in China spanning the period from 2008 to 2022, this study employs a fixed-effects model to unveil the nuanced ways in which mixed ownership influences investment efficiency through the lens of agency costs. This study transcends the boundaries of traditional agency conflicts between managers and shareholders. It delves deeper, illuminating the diverse effects of agency conflicts between significant controlling shareholders and minority shareholders. The results revealed a noteworthy positive correlation between mixed ownership and investment efficiency, and verified the intermediary role of agency costs between mixed ownership and investment efficiency, which is an important result of our research. Heterogeneity analysis indicates that the relationship between the two can be affected by external events, such as during the COVID-19 pandemic, investment efficiency is not the most concerned issue for enterprises. The findings have practical implications for practitioners and policymakers, as they offer avenues for optimizing investment strategies and fostering efficient and effective corporate governance practices.

## 1.Introduction

Mixed ownership has recently emerged as a topic of significant discourse in China. Following market reforms led by socialist principles and the opening of the economy to foreign investors in 1,978, China’s once-stagnant economy has experienced a remarkable revitalization. The relaxation of restrictions on private enterprises has been a pivotal driver of this transformation, propelling the nation through an initial phase of rapid GDP growth and into the current era of sustained high-quality economic development. The contemporary emphasis on reforming mixed-ownership enterprises has garnered considerable attention from practitioners and scholars in the finance field [1]. Distinguishing itself from previous mixed ownership reform initiatives that primarily revolved around financial investment, the present reforms mainly focus on sharing resources and enhancing overall company efficiency [2]. This shift in approach is poised to fulfill the fundamental objective of establishing a modern company framework characterized by diversified equity and equitable distribution of ownership interests.

The investment efficiency of listed companies serves as a reflective indicator of agency issues and a pivotal factor that influences firms’ long-term profitability and sustainable growth [3]. It is widely acknowledged that the inefficiencies observed in state-owned enterprises can be attributed to the principal-agent dilemma [4] and policy encumbrances [5] inherent in the state-ownership framework. The principal-agent relationship in state-owned enterprises has several inherent limitations, including the intricate nature of the principal level, non-market-oriented contractual arrangements, and the transient nature of agency contracts. These contradictions and challenges within the principal-agent relationship have significant repercussions on economic efficiency [6,7]. Moreover, state-owned enterprises bear the burden of diverse social responsibilities. The Chinese government guides the investment behaviors of these enterprises to achieve political objectives, such as fostering social employment and ensuring workers’ welfare. Consequently, enterprises may encounter difficulties solely in pursuing their efficiency objectives as they contend with multifaceted societal considerations [8].

State-owned enterprises play a pivotal role in maintaining China’s socioeconomic stability. Beyond their pursuit of maximizing corporate value, they bear the responsibility of advancing social welfare [9]. However, their actions can result in higher labor adjustment costs compared to non-state firms [10]. Driven by their duty to safeguard the public good, state-owned enterprises possess the authority and capacity to intervene in microenterprise operations. They may amplify investment expenditures in alignment with government mandates, fulfill societal obligations, and adhere to performance evaluation criteria. Conversely, state-owned enterprises may curtail their investments to avert financial challenges and allocate financial resources to alternative objectives. This underscores the significant divergence in the business objectives and functions of state-owned and non-state-owned enterprises. The multifaceted roles and commitments of state-owned enterprises distinguish them from privately owned enterprises, resulting in distinct decision-making approaches and resource allocation strategies.

Moreover, the persistent absence of enticing incentive packages for managers remains a long-standing challenge for China’s state-owned enterprises. In the absence of such incentives, managers may lean toward investing in low-risk and short-term ventures to safeguard their personal interests [11]. Consequently, a notable asymmetry of interests emerges between shareholders and managers, giving rise to Type I agency conflicts. These conflicts ultimately contribute to diminished investment efficiency [12]. Addressing these agency conflicts and establishing suitable incentive mechanisms for managers to align their objectives with those of shareholders and the company’s long-term sustainable development are imperative for enhancing the performance and efficacy of state-owned enterprises.

Furthermore, in China, it is common for companies to have a highly concentrated proportion of state-owned ownership, in which controlling shareholders have a significant incentive to maintain control rights while separating them from cash flow rights. This scenario introduces the potential for tunneling behavior by controlling shareholders, who might seek to extract benefits at the expense of minority shareholders, giving rise to Type II agency conflicts. This tunneling behavior can have detrimental implications for corporations’ long-term investment decisions, ultimately declining investment efficiency. When the controlling shareholders prioritize their personal interests over the overall health of the company, it can lead to a suboptimal allocation of resources and hinder the company’s capacity to make sustainable and strategic investments.

Prior research placed significant emphasis on the concept of agency costs. The pioneering work by Jensen and Meckling [13] highlighted that agency problems stem from the misalignment and incongruence of interests between managers and shareholders. Furthermore, Berle and Means [14] articulated a seminal notion in the realm of state-owned enterprises that the primary agency challenge is not merely the failure of professional managers to serve minority shareholders’ interests, but rather the expropriation of minority shareholders by controlling shareholders [15]. These agency dilemmas can lead to investment inefficiencies, particularly regarding project selection. Notably, non-state-owned shareholders integrated into the equity structure and corporate governance can play a constructive role in mitigating agency costs and enhancing investment efficiency [16]. By actively participating in decision-making processes and advocating for improved corporate governance practices, shareholders can align managerial interests with those of all other shareholders, thereby fostering more effective and efficient investment strategies.

This study makes a valuable contribution to the existing literature on mixed ownership and its impact on investment efficiency. It specifically examines how mixed ownership structures influence investment efficiency and sheds light on various corporate governance issues. This research highlights the importance of further investigating the mechanisms and mediating factors through which mixed-ownership reforms influence state-owned enterprises’ investment efficiency. Moreover, this study proposes practical suggestions for effectively enhancing the investment efficiency of China’s state-owned enterprises.

**Fig 1:**
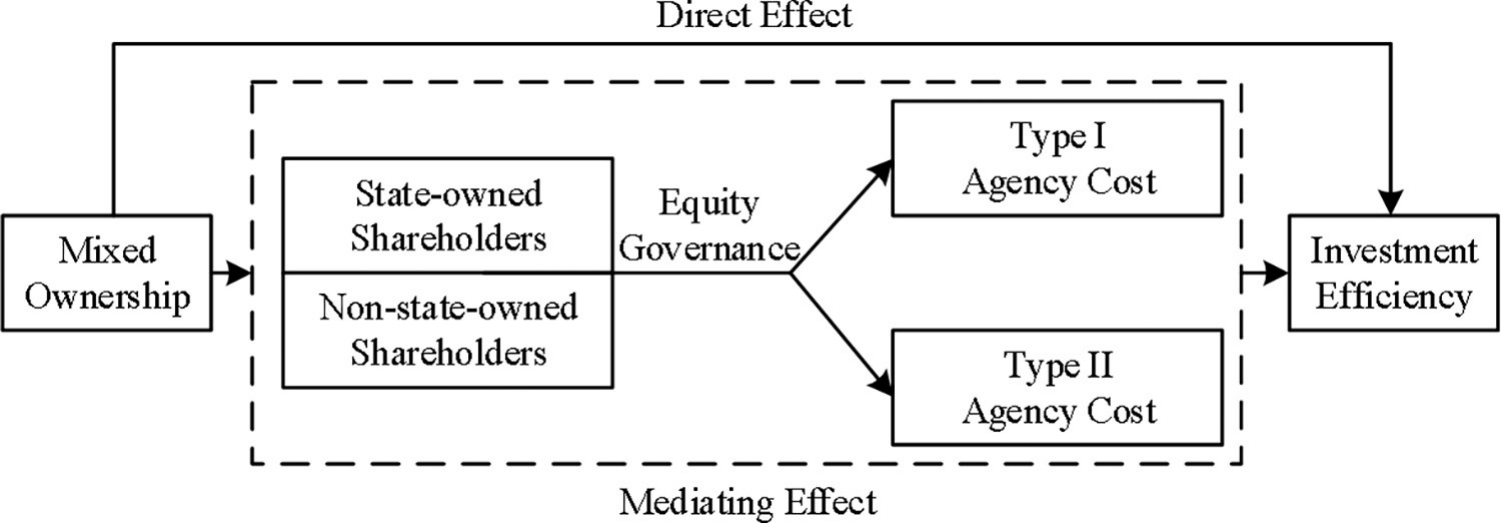
Mechanism of mixed ownership on investment efficiency.

Drawing from principal-agent theory, this study identifies the existence of significant dual agency conflicts within state-owned enterprises in China. Our findings contribute to our understanding of mixed ownership and investment efficiency and emphasize the need for improved corporate governance practices in state-owned enterprises. By addressing agency conflicts and fostering a better alignment of interests among shareholders, state-owned enterprises in China can enhance their investment efficiency and achieve sustainable growth. Further exploration of the influence mechanisms and mediating factors will undoubtedly contribute to the ongoing efforts to reform and improve state-owned enterprises’ investment efficiency in China. A design diagram of the mechanism used in this study is shown in Fig 1.

Fig 1 illustrates the direct and indirect effects of mixed ownership on investment efficiency. This study examines whether mixed ownership positively influences firms’ investment efficiency in China’s capital market. We investigated whether the extent of non-state-owned shareholders’ participation is linked to improvements in state-owned enterprises’ investment efficiency while accounting for observable financial and governance factors that directly affect investment efficiency. The findings revealed a significant positive correlation between the degree of mixed-ownership reform and the investment efficiency of state-owned enterprises. This finding indicates that the introduction of private ownership has a beneficial effect on investment efficiency in state-owned enterprises. Furthermore, this study examines the underlying reasons for the impact of mixed ownership on investment efficiency. This verifies the mediating role of agency costs in the relationship between mixed ownership and investment efficiency. This finding suggests that agency costs play a pivotal role in explaining the influence of mixed ownership influences investment efficiency in state-owned enterprises.

The remainder of this paper is organized as follows. Section 2 presents the literature review and hypothesis development. Section 3 presents the data and variable measurement. Sections 4, 5, 6, 7, and 8 present the results, supplemental analyses, discussions, robustness checks, and heterogeneity analysis, respectively. Finally, the theoretical implications, practical implications, limitations and future research directions, and conclusions are presented in Sections 9, 10, 11, and 12, respectively.

## 2. Literature review and hypothesis development

### 2.1 Mixed ownership and investment efficiency

The introduction of non-state-owned capital in mixed ownership reform optimizes the governance structure, fosters resource sharing, and improves the overall operating efficiency of state-owned enterprises [17]. The resource-based theory suggests that enterprises require abundant resources during their development stages. Shareholder diversification through mixed ownership brings various resources, which is crucial for expanding enterprise capabilities. The participation of non-state shareholders enriches the pool of enterprise resources and enhances decision-making efficiency [18]. Diversifying shareholders through mixed ownership is beneficial for meeting the capital requirements of enterprises. Conversely, non-state shareholders often possess a keen market sense and strong inclination towards innovation, which can help enterprises overcome operational challenges and resource limitations [19]. By introducing non-state shareholders, companies can achieve two important objectives: acquiring diverse resources and enhancing their operational efficiency and sustainability [20]. Different shareholders contribute distinct resource clusters to the enterprises. This diversity in resources enables them to sensibly identify and seize investment opportunities during strategic decision-making, effectively reducing risks and uncertainties, and make rational choices [21].

The systematic implementation of mixed ownership reforms has led to a deeper alignment of interests among diverse shareholders. Non-state shareholders, who prioritize self-interested returns, are motivated to enhance corporate governance levels [22,23]. This commitment to improving governance has significant implications for state-owned enterprises, as it helps alleviate their conservative investment tendencies resulting from long-term government intervention [24]. Additionally, it addresses the over-investment issue caused by the state’s preference for allocating resources toward state-owned enterprises [25]. By paying greater attention to maximizing their returns, non-state shareholders in mixed ownership structures advocate for enhanced corporate governance practices. Improved governance can lead to greater transparency, accountability, and better monitoring of management decisions. This can help reduce the conservative approach often adopted by state-owned enterprises owing to historical government involvement.

State-owned enterprises often face significant principal-agent problems resulting from challenges in managerial supervision and inadequate incentive mechanisms. Increasing the proportion of non-state-owned shareholders can be an effective way to address these issues and improve enterprises’ investment activities [26]. This infusion of non-state ownership can have several positive effects on the functioning of state-owned enterprises. First, increasing the presence of non-state-owned shareholders can reduce government intervention in the investment activities of state-owned enterprises and alleviate the burden on policies. This can lead to clearer and more market-oriented business objectives, enabling state-owned enterprises to make investment decisions based on economic viability and strategic goals. Second, non-state-owned shareholders’ active involvement and supervision can serve as a check on the investment activities of managers seeking control rights. This increased supervision can reduce information asymmetry between shareholders and managers, leading to effective and informed investment decisions [27]. Consequently, introducing non-state-owned shareholders in state-owned enterprises represents a strategic approach for achieving an organic combination of mechanisms and resources. It helps address the shortage of resources in state-owned enterprises, enhances corporate governance practices, improves investment capabilities, and overcomes the development challenges faced by state-owned enterprises [28]. Therefore, Hypothesis 1 is proposed.

**Hypothesis 1.** There is a positive relationship between mixed ownership and investment efficiency.

### 2.2 Influencing mechanism(s)

The issues of multi-level principal-agent relationships and the absence of active ownership in state-owned enterprises in China contribute to widespread opportunism and self-interested behavior among senior executives in these enterprises [6]. The lack of effective oversight and monitoring by owners allows senior executives to act in their own self-interest rather than aligning their actions with the best interests of the company and its shareholders. Furthermore, the pursuit of political promotion by state-owned enterprise executives can lead to a positive tendency to defer political will and bear the additional policy burdens imposed by the government. This can result in a misalignment between the investment decisions made by state-owned executives and the financial goals of the company [29]. When the focus shifts from maximizing financial performance to efficiency, it can lead to suboptimal investment decisions and hinder the overall investment efficiency of state-owned enterprises. Agency costs between managers and shareholders are a significant contributing factor to the low investment efficiency of many state-owned enterprises in China. Agency costs arise from conflicts of interest between managers who may prioritize their own interests and shareholders who seek to maximize returns and long-term value. These conflicts can lead to inefficient resource allocation, mismanagement of funds, and lack of focus on achieving the company’s financial objectives.

Principal-agent conflicts between managers and shareholders arise because of unequal access to information. As shareholder agents, managers may possess more information about a company’s operations and financial status, giving rise to potential conflicts of interest. Additionally, serious conflicts can occur between the controlling and minority shareholders [7]. In a limited investor protection system, controlling shareholders may have greater control over a company than their proportionate cash flow rights. This concentrated control gives them the ability to influence corporate decisions and actions, thereby increasing their potential to encroach on the interests of minority shareholders [30]. Consequently, minority shareholders may face challenges in safeguarding their rights and ensuring that their interests are adequately represented in decision-making processes. Such conflicts can impact corporate governance and investment efficiency, particularly in mixed ownership structures, where the interests of different shareholder groups may diverge. Addressing these conflicts is crucial for promoting transparency, accountability, and sustainable growth among listed companies.

With the establishment of mixed ownership reform, the active involvement of non-state capital, driven by economic goals, plays a significant role in mitigating opportunistic behaviors stemming from conflicts between personal and corporate interests. Proactive participation effectively addresses principal-agent conflicts [31,32]. The presence of heterogeneous shareholders enhances supervision and balance within the company because of their diverse risk preferences and interest functions, which in turn places restrictions on the expropriation of minority shareholders by large shareholders [16]. Equity diversification also reduces direct government intervention in enterprise operations. This considerably weakens the political connections between state-owned shareholders and executives, subsequently decreasing the likelihood of collusion between them [26]. Consequently, mixed ownership reform drives an effective governance structure for state-owned enterprises, as it alleviates agency conflicts and improves operating efficiency. The collaborative efforts of state and non-state capital lead to a transparent and accountable corporate environment, fostering the sustainable growth and development for state-owned enterprises in the long run. Therefore, Hypothesis 2 is proposed.

**Hypothesis 2.** Agency costs will have a mediating effect on the relationship between mixed ownership and investment efficiency.

## 3. Data and variable measurement

### 3.1 Data and sample selection

We conducted an analysis using a sample of 9,274 A-share listed companies from the Shanghai and Shenzhen Stock Exchange in China, covering the period from 2008 to 2022. In the regression model, we regress the explanatory variables on the lagged dependent variable. Therefore, the dependent variable of efficiency investment in this study covers data from 2009 to 2022, while the explanatory variables cover data from 2008 to 2021. To ensure data quality and validity, we implemented several screening criteria for unbalanced panel data.

1. Exclusion of samples with missing data: Companies with incomplete or missing data were excluded from the analysis to maintain data integrity and accuracy.
2. Exclusion of samples with an asset liability ratio negative or greater than one: Companies with asset liability ratios outside a reasonable range were removed from the sample to avoid distortions in the results.
3. Exclusion of financial and insurance industries: To focus on non-financial industries, we excluded companies operating in the financial and insurance sectors from our analysis.
4. Exclusion of samples with extreme values: We eliminated samples with extreme values that could unduly influence the study outcomes, ensuring a more reliable and representative dataset.
5. Exclusion of samples with less than one year of listing: Companies with less than one year of listing time were excluded to provide sufficient observation periods for a comprehensive analysis of investment efficiency.

We opted for a one-year lagged panel of data on mixed ownership because we recognize that it takes time for the effects of the mixed ownership reform to manifest and impact investment efficiency. Using a lagged approach, we observe changes in investment efficiency over time and better understand the gradual impact of mixed ownership on the listed companies. Additionally, to address concerns related to endogeneity and simultaneous effects, we selected one-year lagged data as control variables. This approach helps to mitigate any potential biases that could arise from variables influencing each other simultaneously. All data used in our study were sourced from reliable databases, specifically the China Stock Market & Accounting Research Database (CSMAR) and annual reports of listed companies. This ensured the accuracy and credibility of the data used in the analysis. To safeguard against any potential undue influence of extreme values on our research results, we conducted a 1% up-and-down winsorization on all continuous variables. Winsorization involves replacing extreme values with those that are closer to the rest of the data, thus preventing them from disproportionately affecting the overall analysis.

Table 1 presents the definitions of the dependent and explanatory variables used in the analysis.

**Table 1:** Variable definitions.

Variable	Abbreviation	Definition
Inefficient investment	*INVEFF*	The absolute value of regression residuals for Model (1)
Over_investment	*OverInv*	The positive residuals of Model (1)
Under_investment	*UnderInv*	The absolute value of negative residuals for Model (1)
Degree of mixed ownership	*Mixtra*	Total shareholding ratio of non-state-owned shareholders of the top ten shareholders
	*Restri*	The difference between the proportion of non-state shares and the proportion of state shares among the top ten shareholders
Management expense ratio	*Adm*	The log of management expense / total assets
Other Receivables	*Ore*	The log of other receivables / total assets
Company size	*Size*	The natural logarithm of the total book value of assets
Asset-liability ratio	*Lev*	The ratio of the book value of total liabilities / the book value of total assets
Cash Flow Ratio	Cash	The net cash flow generated from operating activities /total assets
Quick ratio	*Quick*	(Current Assets—Inventory) / Current Liabilities.
The proportion of accounts receivable	*REC*	Net Accounts Receivable / Total Assets
Proportion of independent directors	*Ind*	The proportion of the number of independent directors in the total number of directors on the board
Shareholding ratio of the largest shareholder	*Top*	The percentage of common shares owned by the largest shareholder
Executive salary	*Salary*	The natural logarithm of the top three executives’ total remuneration
Equity checks and balances	*Balance*	Percentage of Ownership by the Second Largest Shareholder / Percentage of Ownership by the Largest Shareholder
CEO-chair duality	*Dual*	A dummy variable equal to one when the same person serves as both board chair and CEO and zero when the positions of chair and CEO are separate

Source: Authors Design.

### 3.2 Variable measurement

#### 3.2.1 Investment efficiency

In this study, we designed Model (1) to measure investment efficiency by drawing inspiration from Richardson’s work in 2006 [33] and incorporating insights from a recent research of Wu, You, Wang, and Chan in 2023 [34]. Model (1) is based on a regression framework that allows us to estimate the expected investment level of each company. The residuals obtained from this regression model represent the deviation of the actual investment levels from the expected values.


$${Inv}_{i,t}=\beta_{0}+\beta_{1}{Grow}_{i,t-1}+\beta_{2}{Lev}_{i,t-1}+\beta_{3}{Cash}_{i,t-1}+\beta_{4}{Ret}_{i,t-1}+\beta_{5}{Size}_{i,t-1}+\beta_{6}{Age}_{i,t-1}+\beta_{7}{Inv}_{i,t-1}+\sum Year+\sum Industry+\varepsilon_{i,t}$$ Model(1)

The study defines inefficient investment (*INVEFF*) as the absolute value of the residuals (*ε*_*i*,*t*_) obtained from Model (1), as previously mentioned. These residuals represent the difference between a firm’s actual investment level in year *t* and the expected investment level based on the regression model. Positive residuals (*OverInv*) indicate the existence of over-investment in a firm, meaning that the actual investment is higher than expected. Negative residuals (*UnderInv*) indicate the presence of under-investment in a firm, signifying that the actual investment is lower than expected. To obtain the absolute values, the negative residuals were converted to positive. The variables used to measure *INVEFF* and other control variables are as follows [3,33,35]:

1. *Inv*_*i*,*t*_ and *Inv*_*i*,*t-1*_: These variables represent the firm’s new investments in years *t* and *t-1*, respectively. They are calculated as the ratio of the difference between expenditures and income on fixed, intangible, and other long-term assets to total assets at the beginning of the year.
2. *Grow*_*i*,*t-1*_: Reflects the investment opportunities of firm *i* in year *t-1*, expressed by Tobin’s Q value, a measure of the market value of a company relative to its asset replacement cost.
3. *Lev*_*i*,*t-1*_: Represents the financial leverage level of firm *i* in year *t-1*, calculated as the ratio of total liabilities to total assets.
4. *Cash*_*i*,*t-1*_: The ratio of net cash flow generated from operating activities to total assets for firm *i* in year *t-1*.
5. *Ret*_*i*,*t-1*_: The company’s annual stock return rate in year *t-1*.
6. *Size*_*i*,*t-1*_: The size of a firm, represented by the natural logarithm of total assets in year *t-1*.
7. *Age*_*i*,*t-1*_: The listing age of a firm at the end of year *t-1*, calculated as the difference between the statistical year and IPO year.

Additionally, the study includes Year and Industry dummy variables to control for year- and industry-specific effects on investment efficiency.

#### 3.2.2 Mixed ownership

According to Ma, Wang, and Zhang [36], the degree of mixed ownership is measured from the perspective of micro-capital. The researchers determined the nature and shareholding ratio of the top ten shareholders in the CSMAR database using a combination of software analysis and manual data collection. The identified diverse shareholders of state-owned enterprises are then categorized into five groups: “state-owned shareholders”, “private shareholders”, “foreign shareholders”, “financial shareholders”, and “others”. To assess the impact of non-state-owned shareholders and their role in achieving the full effects of the reforms, we focus on the sum of the shareholding ratios of non-state-owned shareholders among the top ten shareholders. This sum was used as an indicator of the degree of mixed ownership.

#### 3.2.3 Mediating variable: Agency costs

In this study, the researchers distinguished between the two types of agency conflicts based on different indicators. These agency conflicts are Type I and Type II agency costs. Type I agency costs are represented by the management expense ratio, which was introduced by Owusu and Weir in 2018 [37]. Management expense ratio measures the proportion of management expenses to total assets. A higher management expense ratio indicates higher agency costs because it implies that a larger portion of company resources is allocated toward management expenses rather than productive investments or shareholder returns. This suggests potential conflicts between managers and shareholders, in which managers might prioritize their own interests over maximizing shareholder value. Type II agency costs are represented by funds held by major shareholders; specifically, the ratio of other receivables to total assets. Chen et al. [1] explored this indicator. This ratio reflects the amount of funds that major shareholders may hold as receivables from the company. A higher ratio indicates a stronger likelihood of agency conflict between controlling shareholders and minority shareholders. This suggests that major shareholders may use their controlling power to expropriate company resources at the expense of minority shareholders.

#### 3.2.4 Control variables

Consistent with prior research [3,35], we have carefully selected appropriate control variables to examine the impact of mixed ownership on investment efficiency. To ensure the robustness of our findings, we employed various categorization systems for these control variables. The first category focuses on financial attributes including firm size (*Size*), financial leverage (*Lev*), cash flow ratio (*Cash*), quick ratio (*Quick*) and the proportion of accounts receivable (*REC*). These variables were selected based on their relevance to our research question. In the second category, we examined the realm of corporate governance indices. Within this category, we considered the proportion of ownership held by the largest shareholder (*Top*), executive compensation (*Sal*), equity checks and balances (*Balance*), CEO-chair duality (*Dual*) and board independence (*Ind*). These variables are carefully selected to gauge the influence of corporate governance on the objectives of our study. Furthermore, we employed a firm fixed effects model to account for firm characteristics that could impact *Mixtra* and *INVEFF*. For a comprehensive understanding, we provide detailed definitions of all variables in Table 1 to ensure transparency and clarity in our research methodology.

## 4. Results

### 4.1 Descriptive statistics

Descriptive statistical outcomes are presented in Table 2. The comprehensive mean value of *INVEFF* among the registered Chinese companies was 0.018. This mean surpasses the median value of 0.012. Additionally, the standard deviation for this metric was calculated as 0.021, indicating the level of dispersion in the data. Remarkably, approximately 61.47% of the firms encountered instances of under-investment. This substantial proportion underscores the prevalence of under-investment among listed Chinese companies. However, juxtaposing the mean values of under-investment (*UnderInv*) and over-investment (*OverInv*) reveals a considerable pattern of significant over-investment and under-investment issues within China’s listed corporate landscapes.

**Table 2:** Descriptive statistics of main variables.

Variable	Obs	Mean	Median	Std. Dev	Min.	Max.
*INVEFF*	9,274	0.018	0.012	0.021	0	0.196
*OverInv*	3,573	0.024	0.014	0.028	0	0.196
*UnderInv*	5,701	0.015	0.011	0.015	0	0.148
*Mixtra*	9,274	0.321	0.306	0.232	0.009	0.850
*Size*	9,274	22.93	22.81	1.431	20.13	26.46
*Lev*	9,274	0.505	0.516	0.194	0.082	0.887
*Cashflow*	9,274	0.052	0.0510	0.0680	-0.222	0.283
*Quick*	9,274	1.272	0.904	1.445	0.131	33.96
*REC*	9,274	0.091	0.0580	0.0950	0	0.506
*Ind*	9,274	0.371	0.333	0.0560	0.308	0.571
*Top*	9,274	0.395	0.390	0.155	0.106	0.750
*Sal*	9,274	14.46	14.44	0.760	12.42	16.55
*Balance*	9,274	0.283	0.166	0.278	0.006	1
*Dual*	9,274	0.098	0	0.297	0	1

Source: Authors Estimation.

The mean value attributed to the mixed ownership reform (*Mixtra*) was 32.1%, with a median of 30.6%. Notably, the lower limit is only 0.9%. This substantial variation in *Mixtra* underscores the significant divergence in the extent of diversified capital involvement within China’s mixed-ownership reform initiatives. Notably, when observing the sample size, it is evident that the progress of China’s mixed ownership reform remains quite incomplete. Moreover, the mean value of the shareholding ratio of the largest shareholder (*Top*) is 39.5%, and the median is 39%, with the highest reaching 75%. This aligns with the established reality that equity ownership in Chinese listed companies tends to be concentrated.

Moreover, we conducted the pearson correlation analysis of the variables, and the specific results are presented in the Supporting information (Correlation analysis). Notably, the absolute values of these correlation coefficients were < 0.6. This observation suggests that multicollinearity is not prominent within the dataset. Consequently, we can confidently proceed with multiple regression analyses involving the variables.

### 4.2 Changing trend of investment efficiency and mixed ownership

**Fig 2:**
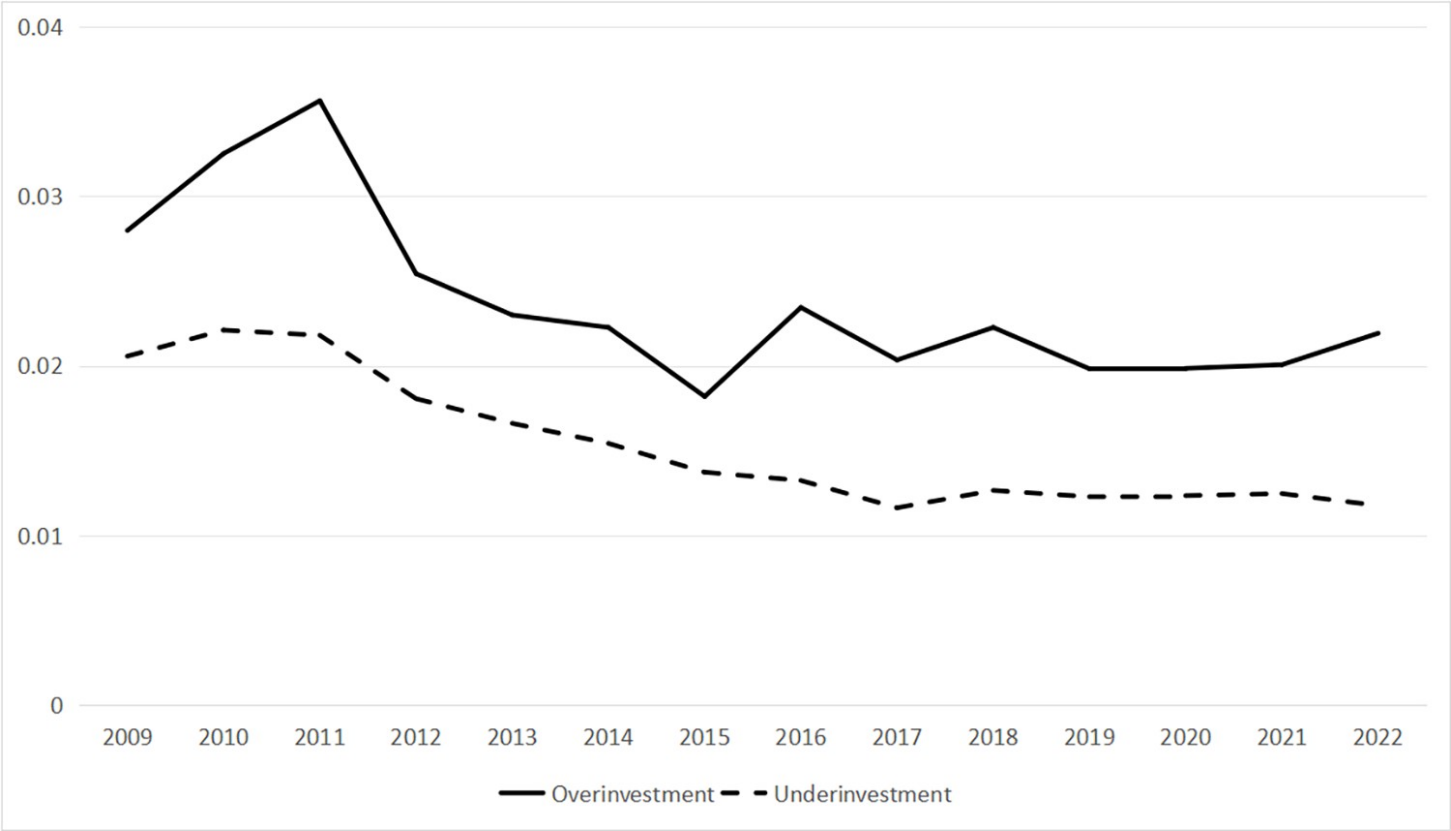
Changing trend of investment efficiency.

Fig 2 is generated using data related to *INVEFF* and illustrates the evolving patterns of annual averages for both over-investment and under-investment within China’s mixed ownership enterprises, spanning the years 2009 to 2022. The curve’s developmental trajectory underscores a general downward trend, lending support to the idea that the overall investment efficiency of Chinese state-owned enterprises is on an upward trajectory. Nevertheless, the plateau observed in recent years also indicates the necessity for further enhancement in the investment efficiency of Chinese state-owned enterprises.

**Fig 3:**
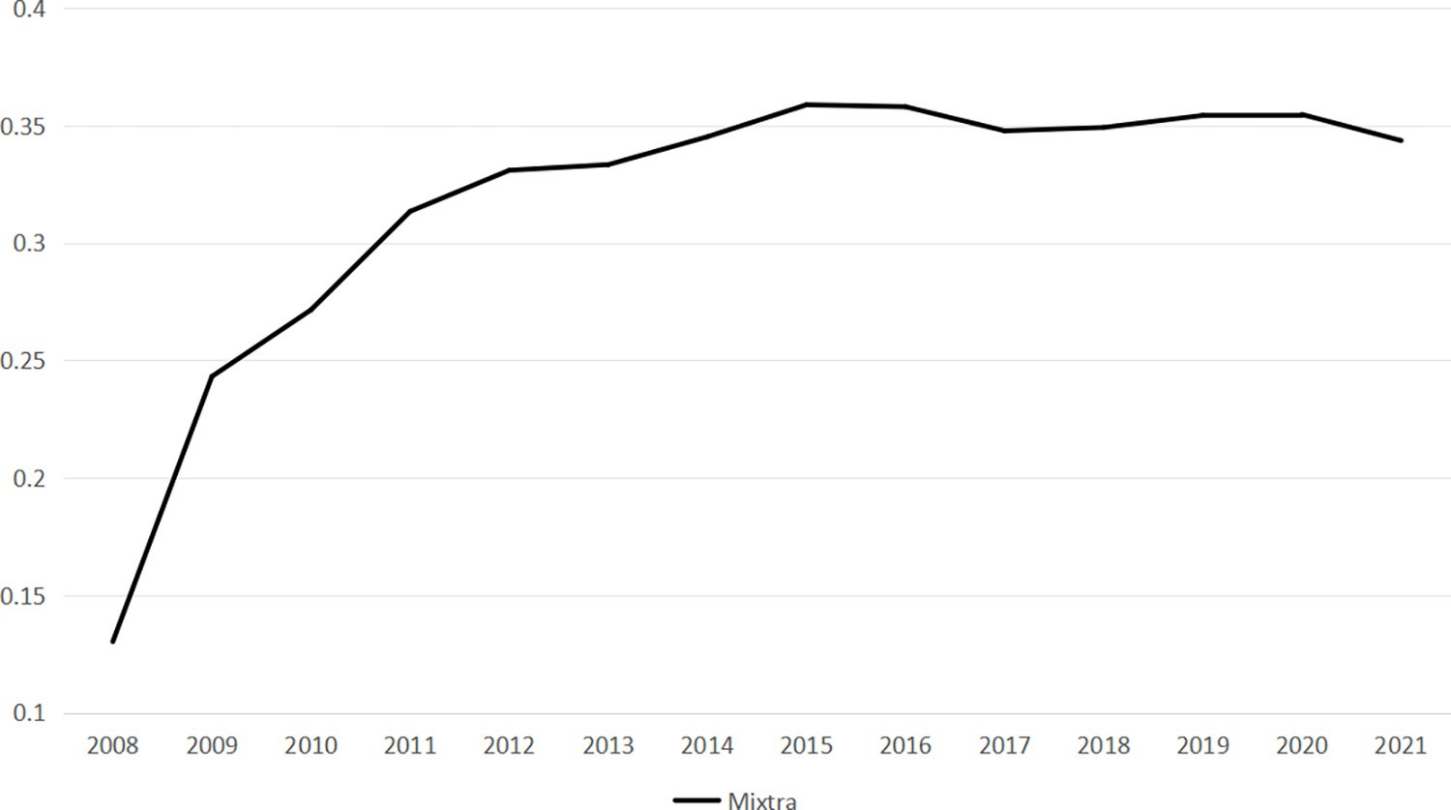
Changing trend of mixed ownership.

Fig 3 depicts the trend in the average shareholding ratios of the top ten non-state shareholders in Chinese mixed-ownership enterprises from 2008 to 2021 using annual average values from *Mixtra* data. The curve exhibits a general upward trend, with a substantial increase between 2008 and 2011, followed by a slow rise from 2011 to 2021, stabilizing within the range of 0.25–0.35. When combined with the descriptive statistical findings, it is clear that over the last decade, the shareholding ratio of non-state shareholders in Chinese mixed-ownership enterprises has concentrated around 30%.

The curve depicted in Figs 2 and 3 underscores a compelling observation. The integration of diverse resources through mixed ownership reform in China has a substantial influence on the enhancement of investment efficiency. This phenomenon represents a valid and effective approach toward the gradual enhancement of corporate governance within China’s enterprises.

### 4.3 Regression analysis

To test Hypothesis 1, which posits a positive relationship between the extent of mixed ownership reform and investment efficiency, we estimate regression Model (2).


$${INVEFF}_{i,t}=\beta_{0}+\beta_{1}{Mixtra}_{i,t-1}+{\beta_{2}{Size}_{i,t-1}+\beta_{3}{Lev}_{i,t-1}+\beta }_{4}{Cash}_{i,t-1}+\beta_{5}{Quick}_{i,t-1}+\beta_{6}{REC}_{i,t-1}+\beta_{7}{Ind}_{i,t-1}+\beta_{8}{Top}_{i,t-1}+\beta_{9}{Sal}_{i,t-1}+\beta_{10}{Balance}_{i,t-1}+\beta_{11}{Dual}_{i,t-1}+\lambda +\varepsilon$$ Model(2)

Here, *INVEFF*_*i*,*t*_ represents the absolute value of the residuals (*ε*_*i*,*t*_) derived from Model (1), indicating inefficient investment for firm *i* during year *t*. A higher value of *INVEFF* denotes lower investment efficiency. Coefficients *β*_*0*_ through *β*_9_ signify the regression coefficients in Model (2), and *ε* denotes a random variable. The control variables incorporated are *Size*, *Lev*, *Cash*, *Quick*, *REC*, *Ind*, *Top*, *Sal*, *Balance* and *Dual*. *Mixtra* represents the extent of mixed ownership. Our estimation utilizes a fixed-effects model approach, *λ* represents the firm fixed effects controlling for the sample. The specific outcomes of the regression Model (2) are shown in columns (1), (2), and (3) of Table 3.

**Table 3:** Mixed ownership and investment inefficiency.

	(1)	(2)	(3)	(4)	(5)
VARIABLES	*INVEFF*	*OverInv*	*UnderInv*	*Adm*	*INVEFF*
*Mixtra*	-0.0031***	-0.0054**	-0.0028**	-0.1125***	-0.0029**
	(-2.60)	(-2.04)	(-2.36)	(-5.07)	(-2.45)
*Adm*					0.0015**
					(2.57)
*Size*	-0.0051***	-0.0064***	-0.0039***	-0.3176***	-0.0046***
	(-11.04)	(-6.24)	(-8.35)	(-36.20)	(-9.28)
*Lev*	-0.0040	-0.0154***	0.0039	0.1034**	-0.0042*
	(-1.61)	(-2.72)	(1.58)	(2.19)	(-1.67)
*Cashflow*	0.0117***	0.0172**	0.0071**	-0.0387	0.0117***
	(3.28)	(2.03)	(2.04)	(-0.58)	(3.30)
*Quick*	0.0001	0.0010	-0.0006***	-0.0158***	0.0001
	(0.46)	(1.60)	(-2.80)	(-3.46)	(0.55)
*REC*	-0.0132**	0.0001	-0.0206***	0.6806***	-0.0142***
	(-2.56)	(0.01)	(-4.22)	(6.99)	(-2.75)
*Ind*	-0.0134**	-0.0145	-0.0086	-0.2729***	-0.0130**
	(-2.45)	(-1.17)	(-1.59)	(-2.64)	(-2.37)
*Top*	0.0018	-0.0014	0.0020	0.4888***	0.0011
	(0.45)	(-0.16)	(0.51)	(6.54)	(0.27)
*Sal*	-0.0005	0.0009	-0.0011**	-0.0229**	-0.0004
	(-0.83)	(0.69)	(-2.00)	(-2.13)	(-0.76)
*Balance*	0.0007	0.0009	-0.0006	0.0482	0.0007
	(0.44)	(0.25)	(-0.38)	(1.52)	(0.39)
*Dual*	-0.0005	-0.0000	-0.0006	-0.0261	-0.0005
	(-0.58)	(-0.01)	(-0.74)	(-1.62)	(-0.53)
*Constant*	0.1501***	0.1701***	0.1249***	4.0628***	0.1440***
	(17.99)	(9.21)	(14.77)	(25.77)	(16.58)
*Firm fixed effects*	Yes	Yes	Yes	Yes	Yes
*Observations*	9,274	3,573	5,701	9,274	9,274
*R-squared*	0.040	0.043	0.051	0.261	0.041
*Number of id*	1,235	1,003	1,171	1,235	1,235

Note

***p < 0.01

**p < 0.05

*p < 0.1.

Source: Authors Estimation.

The first column of Table 3 provides insights into the impact of mixed ownership on investment inefficiency. Notably, the regression coefficient pertaining to mixed ownership and inefficient investment was -0.0031 for the full sample. This coefficient exhibits a significant negative correlation at the 1% level of statistical significance. This negative correlation signifies that as mixed ownership reforms become more deeply embedded, investment efficiency is notably enhanced. Thus, the confirmation of this outcome aligns with and validates Hypothesis 1 as postulated in this study.

The control variables outlined exhibit significant relationships with *INVEFF*. We find a significant negative correlation between firm size and investment inefficiency. This suggests that larger-scale mixed ownership firms tend to have higher investment efficiency. This relationship implies that as the size of a company increases, it may have better resources and capabilities to optimize its investment decisions. The cash flow ratio exhibits a significant positive correlation with inefficient investment, indicating that higher cash flows may potentially decrease corporate investment efficiency. The proportion of accounts receivable shows a significant negative correlation with inefficient investment, signifying that a higher proportion of accounts receivable, coupled with relatively lower cash flow, can effectively reduce corporate inefficient investment. Additionally, we find that an increase in the proportion of independent directors contributes to curbing inefficient corporate investments.

To evaluate Hypothesis 2, which postulates the existence of a mediating effect of agency costs on the connection between mixed ownership and investment efficiency, we estimate regression Models (3) and (4).


$${Adm}_{i,t-1}=\beta_{0}+\beta_{1}{Mixtra}_{i,t-1}+{\beta_{2}{Size}_{i,t-1}+\beta_{3}{Lev}_{i,t-1}+\beta }_{4}{Cash}_{i,t-1}+\beta_{5}{Quick}_{i,t-1}+\beta_{6}{REC}_{i,t-1}+\beta_{7}{Ind}_{i,t-1}+\beta_{8}{Top}_{i,t-1}+\beta_{9}{Sal}_{i,t-1}+\beta_{10}{Balance}_{i,t-1}+\beta_{11}{Dual}_{i,t-1}+\lambda +\varepsilon$$ Model(3)

$${INVEFF}_{i,t}=\beta_{0}+\beta_{1}{Mixtra}_{i,t-1}+\beta_{2}{Adm}_{i,t-1}+{\beta_{3}{Size}_{i,t-1}+\beta_{4}{Lev}_{i,t-1}+\beta }_{5}{Cash}_{i,t-1}+\beta_{6}{Quick}_{i,t-1}+\beta_{7}{REC}_{i,t-1}+\beta_{8}{Ind}_{i,t-1}+\beta_{9}{Top}_{i,t-1}+\beta_{10}{Sal}_{i,t-1}+\beta_{11}{Balance}_{i,t-1}+\beta_{12}{Dual}_{i,t-1}+\lambda +\varepsilon$$ Model(4)

In the context of firm *i* and year *t*, the focal point of Model (3) pertains to the management expense ratio (*Adm*), which serves as the dependent variable. This ratio is a representative measure of agency costs. The control variables used in Models (3) and (4) remain consistent with those in Model (2).

The assessment of the mediating effect involved a systematic approach that encompassed three distinct stages. In the initial step, we estimate regression Model (2) to ascertain the potential presence of a markedly negative impact of mixed ownership (*Mixtra*) on *INVEFF*. Subsequently, our focus shifts to the second step, wherein we undertake the estimation of regression Model (3) to elucidate the extent of the significant association between mixed ownership (*Mixtra*) and agency costs (*Adm*). After completing the first two stages, we proceed to the third step. In Model (4), with *INVEFF* as the dependent variable, the independent variables are *Adm* and *Mixtra*. Our scrutiny is directed towards discerning the significance of the regression coefficient. If the *β*_*1*_ coefficient in Model (3) and the *β*_*2*_ coefficient in Model (4) emerge as statistically significant, our attention turns towards a meticulous observation of the coefficient *β*_*1*_ within Model (4) to derive a comprehensive judgment. These intricate regression outcomes are detailed in columns (1), (4), and (5) of Table 3.

Table 3 presents compelling evidence indicating the noteworthy impact of mixed ownership on the enhancement of investment efficiency. The findings displayed in column (4) of the regression results substantiate a significant and negative correlation between *Mixtra* and *Adm*. Through the infusion and amalgamation of non-state-owned capital, a gradual reduction in agency costs becomes evident. By concurrently considering the influence of *Mixtra* and *Adm* within the framework of Model (4), the regression outcomes showcased in column (5) reveal a slight decrease in the magnitude of the regression coefficient linking mixed ownership and inefficient investment, diminishing from 0.0031 to 0.0029. Remarkably, it is discernible that coefficients *β*_*1*_ within Model (4) exhibit negative correlations with *INVEFF*, and *β*_*2*_ exhibit positive correlations with *INVEFF*. Drawing upon the mediating effect test procedures and methodologies outlined by Wen, Zhang, Hou, and Liu [38], it is evident that the reform involving mixed ownership effectively achieves the objective of augmenting investment efficiency by progressively curbing agency costs. Notably, agency costs emerge as a partial intermediary factor within this dynamic. Thus, Hypothesis 2 can be affirmed as supported by the empirical findings.

## 5. Supplemental analyses

To provide a deeper understanding of the mediating role of agency costs between mixed ownership and investment efficiency, this study takes a nuanced approach by distinguishing between two distinct categories of agency costs and conducting supplementary analyses. The principal aim of mixed ownership reform is to enhance corporate governance efficiency [39]. Nevertheless, the concentration of equity can trigger conflicts among shareholders, while its dispersion can give rise to intricate principal-agent challenges between shareholders and managers. These conflicts extend beyond the conventional realm of principal-agent dynamics between shareholders and managers, encompassing conflicts between controlling and minority shareholders [7]. Consequently, the landscape of mixed-ownership enterprises is marked by the coexistence of dual-agency conflicts.

### 5.1 Type I agency cost

In modern enterprises, the fundamental source of Type I agency conflicts lies in the division between management authority and ownership. According to agency theory, managers are prone to opportunistic conduct, exemplified by excessive investments aimed at securing heightened compensation and political advancement [40], as well as insufficient investments aimed at mitigating financial constraints. As the reform of state-owned enterprises progressed, the proportion of shares held by non-state-owned shareholders gradually increased. This shift alleviates the predicament arising from the ambiguous property rights associated with state-owned enterprises.

Furthermore, the growing ownership share of non-state shareholders has prompted vigorous efforts to establish effective incentives and control mechanisms to maximize investment returns. These initiatives counteract managerial moral hazards triggered by information asymmetry, alleviate managerial complacency, and harmonize managerial motivations with shareholder interests. In this context, the supervisory influence exerted by non-state shareholders emerges as a means of mitigating principal-agent conflict, thereby potentially contributing to an improvement in investment efficiency.

### 5.2 Type II agency cost

China’s state-owned listed companies often have concentrated shareholding structures. This concentration has raised concerns regarding the potential adverse repercussions stemming from tunneling effects initiated by controlling shareholders [41]. Type II agency costs are discernible in interactions between controlling and minority shareholders. In the absence of a robust external governance system, a balanced ownership structure assumes pivotal significance in curbing the rent-seeking tendencies and inefficient investment behaviors exhibited by major shareholders. Disparities in interest and risk preferences make heterogeneous shareholders more adept at vigilance and oversight. The ongoing progression of mixed-ownership reform serves to optimize internal governance mechanisms and decentralize equity. This multifaceted approach effectively mitigates self-interested behaviors perpetrated by controlling shareholders and managers, which have historically led to their mutual alignment and detriment to minority shareholders’ interests. Furthermore, it attenuates the sway exerted by controlling shareholders over fund allocation, consequently enhancing enterprises’ investment processes [42].

## 6. Discussion

This study delves into the intricate interplay between mixed ownership and investment efficiency. A central focus was placed on comprehending how mixed ownership shapes investment efficiency dynamics, with particular emphasis on the role of agency costs as potential mediators.

As in the previous study, this study initially identified the management expense ratio (*Adm*) as a constructive proxy indicator for Type I agency costs. Adm represents the ratio of management expenses relative to total assets. Additionally, capital vested in the hands of major shareholders (*Ore*) was chosen as an alternative measure of Type II agency costs [43]. Ore is the ratio of other receivables to total assets.

Moreover, the study acknowledges the influence of the top ten shareholders on a company’s decision-making. To encapsulate this effect, a metric is derived from the difference between the proportion of non-state shares and state shares among the top ten shareholders, an approach rooted in prior scholarly investigations [7]. This metric, termed the "*Restri*", serves as an indicator of the equilibrium achieved among diverse shares and, consequently, offers insight into the extent of mixed ownership reform.

Subsequently, our examination delves into the realms of Models (5), (6), and (7) in a concerted effort to dissect the ramifications of mixed ownership on investment efficiency. Furthermore, we scrutinized the mediating influence of dual-agency conflicts within the intricate interplay between these variables. Retaining the definitions articulated earlier, the other variables remained consistent across the models. The analytical journey undertaken in Models (5), (6), and (7) seeks to unravel the intricate mechanisms underlying the impact of mixed ownership on investment efficiency, with a dedicated focus on the intermediary role of dual agency conflicts within this intricate relationship.


$${INVEFF}_{i,t}=\beta_{0}+\beta_{1}{Restri}_{i,t-1}+{\beta_{2}{Size}_{i,t-1}+\beta_{3}{Lev}_{i,t-1}+\beta }_{4}{Cash}_{i,t-1}+\beta_{5}{Quick}_{i,t-1}+\beta_{6}{REC}_{i,t-1}+\beta_{7}{Ind}_{i,t-1}+\beta_{8}{Top}_{i,t-1}+\beta_{9}{Sal}_{i,t-1}+\beta_{10}{Balance}_{i,t-1}+\beta_{11}{Dual}_{i,t-1}+\lambda +\varepsilon$$ Model(5)

$${Adm}_{i,t}({Ore}_{i,t-1})=\beta_{0}+\beta_{1}{Restri}_{i,t-1}+{\beta_{2}{Size}_{i,t-1}+\beta_{3}{Lev}_{i,t-1}+\beta }_{4}{Cash}_{i,t-1}+\beta_{5}{Quick}_{i,t-1}+\beta_{6}{REC}_{i,t-1}+\beta_{7}{Ind}_{i,t-1}+\beta_{8}{Top}_{i,t-1}+\beta_{9}{Sal}_{i,t-1}+\beta_{10}{Balance}_{i,t-1}+\beta_{11}{Dual}_{i,t-1}+\lambda +\varepsilon$$ Model(6)

$${INVEFF}_{i,t}=\beta_{0}+\beta_{1}{Restri}_{i,t-1}+\beta_{2}{Adm}_{i,t-1}({Ore}_{i,t-1})+{\beta_{3}{Size}_{i,t-1}+\beta_{4}{Lev}_{i,t-1}+\beta }_{5}{Cash}_{i,t-1}+\beta_{6}{Quick}_{i,t-1}+\beta_{7}{REC}_{i,t-1}+\beta_{8}{Ind}_{i,t-1}+\beta_{9}{Top}_{i,t-1}+\beta_{10}{Sal}_{i,t-1}+\beta_{11}{Balance}_{i,t-1}+\beta_{12}{Dual}_{i,t-1}+\lambda +\varepsilon$$ Model(7)

Columns (1), (3), and (5) of Table 4 distinctly portray a significant and negative correlation between the degree of mixed ownership and inefficient investment. The affirmation of Hypothesis 1 was supported by these findings. A deeper analysis, as evident from the outcomes presented in Columns (2) and (4) of Table 4, underscores the multifaceted impact of mixed ownership, elucidating its potential to curtail both Type I and Type II agency costs.

**Table 4:** Test results of the mediating effect of two types of agency costs.

	(1)	(2)	(3)	(4)	(5)
VARIABLES	*INVEFF*	*Adm*	*INVEFF*	*Ore*	*INVEFF*
*Restri*	-0.0019***	-0.0670***	-0.0018***	-0.1050***	-0.0018***
	(-3.22)	(-6.05)	(-3.04)	(-3.82)	(-3.13)
*Adm*			0.0015**		
			(2.50)		
*Ore*					0.0005**
					(2.03)
*Size*	-0.0051***	-0.3183***	-0.0047***	-0.0286	-0.0051***
	(-11.09)	(-36.36)	(-9.34)	(-1.32)	(-11.06)
*Lev*	-0.0039	0.1090**	-0.0040	1.0783***	-0.0044*
	(-1.55)	(2.31)	(-1.61)	(9.23)	(-1.75)
*Cash*	0.0116***	-0.0403	0.0117***	-0.4701***	0.0118***
	(3.26)	(-0.60)	(3.28)	(-2.82)	(3.33)
*Quick*	0.0001	-0.0159***	0.0001	-0.0123	0.0001
	(0.44)	(-3.49)	(0.54)	(-1.09)	(0.46)
*REC*	-0.0132**	0.6820***	-0.0142***	1.5275***	-0.0139***
	(-2.55)	(7.01)	(-2.74)	(6.33)	(-2.69)
*Ind*	-0.0132**	-0.2659***	-0.0128**	0.0962	-0.0132**
	(-2.41)	(-2.58)	(-2.34)	(0.38)	(-2.42)
*Top*	-0.0001	0.4237***	-0.0007	0.1600	-0.0001
	(-0.01)	(5.59)	(-0.17)	(0.85)	(-0.03)
*Sal*	-0.0004	-0.0217**	-0.0004	-0.1793***	-0.0003
	(-0.76)	(-2.02)	(-0.70)	(-6.75)	(-0.60)
*Balance*	0.0001	0.0271	0.0001	0.2474***	0.0000
	(0.09)	(0.85)	(0.06)	(3.14)	(0.01)
*Dual*	-0.0005	-0.0262	-0.0005	-0.0588	-0.0005
	(-0.59)	(-1.63)	(-0.54)	(-1.47)	(-0.55)
*Constant*	0.1498***	4.0545***	0.1438***	-2.3919***	0.1509***
	(17.98)	(25.78)	(16.60)	(-6.13)	(18.08)
*Firm fixed effects*	Yes	Yes	Yes	Yes	Yes
*Observations*	9,274	9,274	9,274	9,274	9,274
*R-squared*	0.040	0.262	0.041	0.038	0.041
*Number of id*	1,235	1,235	1,235	1,235	1,235

Note

***p < 0.01

**p < 0.05

*p < 0.1.

Source: Authors Estimation.

Comparing the outcomes in Column (3) with those in Column (1) of Table 4, it is evident that the absolute value of the *Restri* coefficient has diminished. Furthermore, when juxtaposing the results in Column (5) with those in Column (1) of Table 4, a similar trend is observed, with the absolute value of the regression coefficient of *Restri* decreasing from 0.019 to 0.018. These dynamic underscores the pivotal role of ownership diversity in elevating investment efficiency by mitigating the agency conflicts that permeate the relationship between shareholders and managers, encompassing both controlling and minority shareholders.

Both *Restri* and *Adm/Ore* coefficients are statistically significant, amplifying the robustness of these findings. Drawing on the mediating effect test approach outlined by Wen et al. [38], the collective evidence points towards mixed ownership’s substantial capacity to enhance investment efficiency through the dual reduction of Type I and Type II agency costs.

## 7. Robustness checks

### 7.1 Replacement indicators (Mixtra)

Building on the groundwork of Luo et al. [42], our investigation delved into a similar empirical assessment to quantify mixed ownership by utilizing the difference between the proportion of non-state shares and state shares among the top five shareholders. The outcomes of this analysis, presented in Table 5, align conspicuously with earlier empirical findings, thereby establishing a noteworthy degree of consistency.

**Table 5:** Replacement of explanatory variables indicators.

	(1)	(2)	(3)	(4)	(5)
VARIABLES	*INVEFF*	*Adm*	*INVEFF*	*Ore*	*INVEFF*
*Restri*	-0.0016**	-0.2254***	-0.0014*	-0.0671*	-0.0016**
	(-2.07)	(-14.73)	(-1.70)	(-1.77)	(-2.03)
*Adm*			0.0012*		
			(1.68)		
*Ore*					0.0005*
					(1.87)
*Constant*	0.1243***	3.0278***	0.1207***	-2.5278***	0.1256***
	(10.96)	(13.71)	(10.46)	(-4.63)	(11.06)
*Control*	Yes	Yes	Yes	Yes	Yes
*Firm fixed effects*	Yes	Yes	Yes	Yes	Yes
*Observations*	6,480	6,480	6,480	6,480	6,480
*R-squared*	0.030	0.282	0.031	0.036	0.031
*Number of id*	1,109	1,109	1,109	1,109	1,109

Note

***p < 0.01

**p < 0.05

*p < 0.1.

Source: Authors Estimation.

The congruence between these results and the preceding empirical conclusions lends further weight to the robustness and reliability of our study. This substantiates the enduring validity of our earlier assertions about the intricate interplay between mixed ownership, agency costs, and investment efficiency. This collective body of evidence bolsters the foundational insights laid out in this study and contributes to a comprehensive and substantiated understanding of the relationships examined.

### 7.2 Excluding the sample of moderate investments

Adhering to the methodology outlined by Zhou [44] and Chen and Xie [45], our study adopted a similar approach to classify firms exhibiting tendencies towards over-investment and under-investment into ten distinct groups. The two groups with the smallest residuals were excluded from the analysis. This meticulous curation resulted in a streamlined regression sample size of 8,347.

The empirical findings stemming from this refined approach are presented in Table 6. Evidently, these outcomes reaffirm the central proposition that mixed ownership plays a contributory role in bolstering investment efficiency through the reduction of agency costs. This alignment between our empirical results and the proposed hypothesis underscores the vital role of mixed ownership in optimizing investment dynamics within the context of diverse agency conflicts, thus fortifying the robustness of our research insights.

**Table 6:** Excluding the sample of moderate investments.

	(1)	(2)	(3)	(4)	(5)
VARIABLES	*INVEFF*	*Adm*	*INVEFF*	*Ore*	*INVEFF*
*Mixtra*	-0.0031**	-0.1112***	-0.0029**	-0.2103***	-0.0030**
	(-2.43)	(-4.66)	(-2.29)	(-3.56)	(-2.34)
*Adm*			0.0016**		
			(2.50)		
*Ore*					0.0006**
					(2.23)
*Constant*	0.1563***	3.9326***	0.1501***	-2.4833***	0.1577***
	(17.34)	(23.19)	(16.06)	(-5.92)	(17.46)
*Control*	Yes	Yes	Yes	Yes	Yes
*Firm fixed effects*	Yes	Yes	Yes	Yes	Yes
*Observations*	8,347	8,347	8,347	8,347	8,347
*R-squared*	0.041	0.251	0.042	0.037	0.042
*Number of id*	1,220	1,220	1,220	1,220	1,220

Note

***p < 0.01

**p < 0.05

*p < 0.1.

Source: Authors Estimation.

## 8. Heterogeneity analysis

Given that the sample period of this study spans from 2008 to 2022, during which the global COVID-19 pandemic broke out, numerous enterprises encountered operational setbacks. Considering the impact of the pandemic, the investment efficiency of enterprises may have faced substantial disruptions during this period, potentially relegating investment efficiency levels as a secondary performance metric for businesses. As shown in Fig 2, the inefficiency investment curve did not show a significant decrease from 2020 to 2022. Furthermore, the curve for over-investment saw a slight increase, suggesting a minor decline in investment efficiency among Chinese mixed-ownership enterprises over this period. Consequently, we divided the samples into two parts for comparative analysis: before the COVID-19 outbreak and after the COVID-19 outbreak. The results in Table 7 show that the regression results of the degree of mixed ownership and inefficient investment before the COVID-19 outbreak are displayed in columns (1) and (3), while the results during the COVID-19 period are shown in columns (2) and (4). In columns (1) and (3), the *Mixtra* coefficient is -0.0033, which is significant at the 5% statistical level, while the *Restri* coefficient is -0.0021, significant at the 1% statistical level, indicating a positive correlation between mixed ownership and investment efficiency when excluding the impact of the COVID-19 pandemic. However, the results in columns (2) and (4) show that the degree of mixed ownership is not related to investment efficiency, possibly due to external unexpected event that has affected the relationship between mixed ownership and investment efficiency. The outbreak of the COVID-19 pandemic has had a significant impact on the normal production and operation of enterprises, with investment efficiency not being the primary concern for businesses.

**Table 7:** Heterogeneity analysis.

	(1)	(2)	(3)	(4)
	*Before the* *COVID-19* *outbreak*	*After the* *COVID-19* *outbreak*	*Before the* *COVID-19* *outbreak*	*After the* *COVID-19* *outbreak*
VARIABLES	*INVEFF*	*INVEFF*	*INVEFF*	*INVEFF*
*Mixtra*	-0.0033**	-0.0019		
	(-2.33)	(-0.51)		
*Restri*			-0.0021***	-0.0002
			(-3.03)	(-0.09)
*Constant*	0.1768***	0.1309***	0.1760***	0.1288***
	(15.29)	(2.67)	(15.27)	(2.63)
*Control*	Yes	Yes	Yes	Yes
*Firm fixed effects*	Yes	Yes	Yes	Yes
*Observations*	6,959	2,315	6,959	2,315
*R-squared*	0.043	0.024	0.043	0.024
*Number of id*	1,073	935	1,073	935

Note

***p < 0.01

**p < 0.05

*p < 0.1.

Source: Authors Estimation.

## 9. Theoretical implications

This study makes several noteworthy theoretical contributions to the literature. First, it addresses a conspicuous gap in the existing scholarly landscape, as underscored by Xu and Zhou [46], by probing the intricate dynamics through which mixed ownership influences investment efficiency. By introducing the agency cost lens, this study offers a novel perspective on the interrelationships within this context, particularly considering the divergent manifestations of agency costs. While prior investigations have scrutinized the implications of mixed ownership on firm performance, its impact on investment efficiency has remained relatively uncharted within the realm of state-owned enterprise reform. Concurrently, research pertaining to enterprise investment efficiency has predominantly focused on managerial inclinations [46], with limited empirical inquiry into the intricate interplay between investment efficiency and corporate governance. In the noteworthy convergence of these two areas, this study unveils the substantial potential of mixed ownership to profoundly shape investment efficiency within the dynamic milieu of China’s mixed ownership reform. By forging this nuanced bridge between corporate governance and investment efficiency, this study offers an insightful exploration into the far-reaching effects of mixed ownership in a distinctive and compelling manner.

A secondary and equally significant theoretical contribution of this study is its concentrated effort to unravel the intricate dynamics underlying the impact of mixed ownership on investment efficiency. By illuminating the ramifications of equity governance on investment efficiency, this study examines an uncharted dimension. Within this context and considering the intricate agency conflicts at play, this study elucidates agency costs as pivotal intermediary variables. These empirical findings underscore the pivotal role of mixed ownership in enhancing investment efficiency through the systematic attenuation of agency costs. This synthesis of insights establishes a notable expansion of the empirical research landscape on mixed ownership. By carving out this novel trajectory and meticulously exploring the relationship between mixed ownership, agency costs, and investment efficiency, this study extends the boundaries of previous research, ushering in new vistas for understanding the impact of mixed ownership on investment dynamics.

Another substantial theoretical contribution of this study pertains to its potential implications for mitigating agency costs in China’s capital markets. While previous research has predominantly focused on Type I agency costs, this study demonstrates a comprehensive perspective by accounting for the evolving landscape of China’s transition from a centralized to a market economy. This transition ushers in a multifaceted scenario in which principal-agent conflicts between managers and shareholders, ascribed to asymmetric information, coexist alongside pronounced conflicts between controlling and minority shareholders. Recognizing this evolving dynamic, this study offers valuable insights into both Type I and Type II agency costs. This nuanced examination goes beyond the traditional confines of agency cost analysis and sheds light on a comprehensive array of challenges facing China’s capital market in its transformational journey. By addressing these diverse forms of agency costs and their intricate interplay in the capital market, this study extends its reach beyond mere theoretical inquiry by offering pragmatic pathways to enhance corporate governance and ultimately reduce agency costs. Thus, this holistic approach meaningfully contributes to the ongoing discourse on agency costs and management within China’s evolving economic landscape.

In conclusion, this study advances our understanding of the intricate dynamics between mixed ownership and investment efficiency and reveals the precise governance mechanisms that underpin this relationship. Moreover, the study reveals a dual-layer mediation effect through which mixed ownership influences investment efficiency. By meticulously dissecting the mediating roles of Type I and Type II agency costs, this study illuminates the pathways through which mixed ownership, agency conflict, and investment efficiency intersect. This nuanced analysis enhances our understanding of the intricate interactions within the corporate landscape, affording a comprehensive picture of the multifaceted impact of mixed ownership on investment efficiency. This study contributes significantly by providing a comprehensive framework that elucidates the broader relationship and highlights the specific mechanisms and mediating factors that drive the observed effects. This comprehensive perspective extends the boundaries of prior research, offering a holistic understanding of how mixed ownership engenders tangible impacts on investment efficiency, thus enriching the scholarly discourse and providing actionable insights for practitioners and policymakers.

## 10. Practical implications

The implications of the findings from this study for mixed ownership practice are significant and multifaceted, offering practical guidance for policy-makers, shareholders, and managers engaged in or considering mixed ownership reform.

First, it underscores the importance of equity governance in mixed-ownership reform strategies. Acknowledging the potential benefits of diverse capital endowments, reform efforts should foster the participation of shareholders with varying resources. Policymakers should design policies that encourage and facilitate the involvement of diverse capital and emphasize the advantages and positive outcomes associated with heterogeneity.

Second, it highlights the pivotal role non-state shareholders play in governance and oversight. To enhance market confidence in non-state-owned enterprises and ensure their active engagement in mixed-ownership reform, efforts should be directed towards bolstering the voices of non-state shareholders and reinforcing their supervisory capacity. This could strengthen property rights protection mechanisms and create a transparent capital entry and exit framework, thus facilitating the seamless movement of different forms of capital.

Third, this study recommends establishing a market-oriented professional management system. This approach aims to enhance incentive and restraint mechanisms for senior executives in state-owned enterprises, mitigating principal-agent conflicts arising from political influences and power-seeking behaviors. Such conflicts can hinder the effectiveness of the state-owned enterprise reform. Adopting a market-oriented professional management system can help mitigate these challenges and ensure a more efficient and merit-based managerial landscape.

In summary, this study advocates a holistic approach to mixed ownership practices, emphasizing equity governance, the involvement of non-state shareholders, and the implementation of market-oriented managerial systems. By embracing these recommendations, stakeholders can navigate the complexities of mixed-ownership reform more effectively, fostering improved corporate governance, investment efficiency, and overall organizational performance.

## 11. Limitations and future research directions

Although this study provides valuable insights, it has certain limitations that should be acknowledged. These limitations pave the way for opportunities to refine and expand the findings of this study for future research endeavors.

First, the concept of mixed ownership is multifaceted and extends beyond a simple proportion of non-state shareholders. It encompasses aspects such as the diversity of non-state shareholders and the potential for change among the largest shareholders of state-owned enterprises. Future research could delve deeper into these dimensions by exploring how they influence the outcomes of mixed-ownership reform. A more nuanced understanding of its impact on investment efficiency can be achieved by adopting a comprehensive approach to assess mixed ownership levels.

Second, while this study identified both Type I and Type II agency costs as partial mediators, additional mediating variables can be included. Exploring a wider range of variables that could mediate the relationship between mixed ownership and investment efficiency could provide a more comprehensive understanding of the underlying mechanisms. This could lead to a refined understanding of the intricate dynamics shaping investment efficiency in the context of mixed ownership.

Third, this study acknowledges the possibility of omitting correlated control variables. The interaction between an organization’s internal environment and the external capital market can significantly influence the governance effects of mixed ownership. Therefore, future research could explore and incorporate additional external factors to enhance the explanatory power of the model, thus providing a holistic view of how mixed ownership interacts with both internal and external factors to influence investment efficiency.

In conclusion, while this study makes valuable contributions, these limitations offer avenues for future studies to build upon, refine the understanding of the effects of mixed ownership on investment efficiency, and delve into a comprehensive exploration of its multidimensional dynamics.

## 12. Conclusion

This study constitutes a rigorous empirical exploration of the intricate interplay between mixed ownership, investment efficiency, and the mediating role of agency costs. The findings substantiate the significant impact of mixed ownership on investment efficiency. The empirical evidence highlights a statistically significant and positive correlation between mixed ownership and investment efficiency, underscoring the pivotal role of mixed ownership in fostering improved investment dynamics within organizations. This study advances our understanding by illuminating the mediating effect of agency costs on this relationship. They identify agency costs as pivotal intermediary variables that channel the influence of mixed ownership on investment efficiency. The results emphasize the partial mediating effects of Type I and Type II agency costs, shedding light on the intricate mechanisms by which mixed ownership propels its influence. In addition, the heterogeneity analysis in this study also proves that, before the COVID-19 outbreak, mixed ownership has a positive impact on investment efficiency. However, during the COVID-19 pandemic, there is no significant relationship between mixed ownership and investment efficiency, indicating that investment efficiency is impacted by external events and improving investment efficiency is not the top priority for enterprises during this period. This study provides robust empirical insight into the significance of mixed ownership in enhancing investment efficiency. This underscores the dual mediation pathways involving agency costs, and establishes a comprehensive understanding of how these factors collectively shape intricate relationships within the corporate landscape. These findings have practical implications for practitioners and policymakers, as they offer avenues for optimizing investment strategies and fostering efficient and effective corporate governance practices.

## Supporting information

S1 Data (XLSX)

S2 Data (XLSX)

S3 Data (XLSX)

S4 Data Correlation analysis. (XLSX)
